# Barriers to and enablers of type 2 diabetes screening among women with prior gestational diabetes: A qualitative study applying the Theoretical Domains Framework

**DOI:** 10.3389/fcdhc.2023.1086186

**Published:** 2023-02-24

**Authors:** Amelia J. Lake, Amelia Williams, Adriana C. H. Neven, Jacqueline A. Boyle, James A. Dunbar, Christel Hendrieckx, Melinda Morrison, Sharleen L. O’Reilly, Helena Teede, Jane Speight

**Affiliations:** ^1^ School of Psychology, Deakin University, Geelong, VIC, Australia; ^2^ The Australian Centre for Behavioral Research in Diabetes, Diabetes Victoria, Melbourne, VIC, Australia; ^3^ Monash Centre for Health Research and Implementation, Monash Public Health and Preventive Medicine, Monash University, Clayton, VIC, Australia; ^4^ Monash Department of Obstetrics and Gynecology, Monash Health, Clayton, VIC, Australia; ^5^ Deakin Rural Health, School of Medicine, Deakin University, Warrnambool, VIC, Australia; ^6^ Diabetes Australia, Canberra, ACT, Australia; ^7^ School of Exercise & Nutrition Science, Deakin University, Burwood, VIC, Australia; ^8^ UCD Institute of Food and Health, College of Health and Agricultural Sciences, University College Dublin, Dublin, Ireland

**Keywords:** gestational diabetes, type 2 diabetes mellitus, glucose tolerance test, postpartum period, female, behavior therapy, persuasive communication, qualitative

## Abstract

**Introduction:**

Women with previous gestational diabetes mellitus (GDM) are at increased risk of type 2 diabetes (T2D). Guidelines recommend postnatal diabetes screening (oral glucose tolerance test or HbA1c) typically 6-12 weeks after birth, with screening maintained at regular intervals thereafter. Despite this, around half of women are not screened, representing a critical missed opportunity for early identification of prediabetes or type 2 diabetes. While policy and practice-level recommendations are comprehensive, those at the personal-level primarily focus on increasing screening knowledge and risk perception, potentially missing other influential behavioral determinants. We aimed to identify modifiable, personal-level factors impacting postpartum type 2 diabetes screening among Australian women with prior gestational diabetes and recommend intervention functions and behavior change techniques to underpin intervention content.

**Research design and methods:**

Semi-structured interviews with participants recruited via Australia’s National Gestational Diabetes Register, using a guide based on the Theoretical Domains Framework (TDF). Using an inductive-deductive approach, we coded data to TDF domains. We used established criteria to identify ‘important’ domains which we then mapped to the Capability, Opportunity, Motivation–Behavior (COM-B) model.

**Results:**

Nineteen women participated: 34 ± 4 years, 19 ± 4 months postpartum, 63% Australian-born, 90% metropolitan, 58% screened for T2D according to guidelines. Eight TDF domains were identified: ‘knowledge’, ‘memory, attention, and decision-making processes’, ‘environmental context and resources’, ‘social influences’, ‘emotion’, ‘beliefs about consequences’, ‘social role and identity’, and ‘beliefs about capabilities’. Study strengths include a methodologically rigorous design; limitations include low recruitment and homogenous sample.

**Conclusions:**

This study identified numerous modifiable barriers and enablers to postpartum T2D screening for women with prior GDM. By mapping to the COM-B, we identified intervention functions and behavior change techniques to underpin intervention content. These findings provide a valuable evidence base for developing messaging and interventions that target the behavioral determinants most likely to optimize T2D screening uptake among women with prior GDM.

## 1 Introduction

Gestational diabetes mellitus (GDM) affects approximately 13% of births worldwide, and is acknowledged as “the fastest growing type of diabetes in Australia” (1, 2). Women with prior GDM have an eight-fold increased lifetime risk of type 2 diabetes (T2D) compared to women with normoglycemic pregnancies (3). Early and regular postpartum T2D screening is essential as undiagnosed and persistent elevated blood glucose levels (hyperglycaemia) increases risk for adverse health outcomes (4). Australian guidelines recommend T2D screening (currently via an Oral Glucose Tolerance Test or OGTT), 6-12 weeks postpartum and, every 1-3 years thereafter (5). Despite the benefits, only 43-58% of women in Australia complete the OGTT (6), reflecting screening rates worldwide (7, 8).

National and international studies have highlighted that policy, practice, and personal-level factors contribute to low screening uptake. Common policy-level factors include limitations related to public healthcare and lack of consensus on screening guidelines (9). Practice-level factors include healthcare silos, lack of focus on ongoing risk in consultations, and lack of reminder systems (9–11). Systematic reviews of qualitative studies have identified common personal-level screening barriers, including competing demands, lack of practical social support, challenges related to the screening procedure, lack of knowledge and absence of advice from health professionals (12–14). Similar findings are reflected in an Australian context (15–21).

Recommendations to increase screening uptake have primarily focused on initiatives at policy and practice-levels including information provision and reminders (12–14). The limitations of such approaches have been acknowledged (6, 22–24). A key element of best practice personal-level intervention development is explicit use of theoretical approaches to both identify behavioral determinants and targeting them via intervention content underpinned by behavior change techniques (25, 26). To date, there has been a paucity of such research. Thus, in-depth exploration of personal-level behavioral factors influencing women’s screening is warranted, to develop recommendations for persuasive messaging grounded in health behavior change theory.

The Theoretical Domains Framework (TDF) is an example of a theoretical approach to identifying determinants of a defined behaviour. The TDF comprises 14 behavior change domains derived from 33 behavior change theories (27). The TDF has been used to identify determinants of health behaviors, including among women with GDM (28).

**Figure 1:**
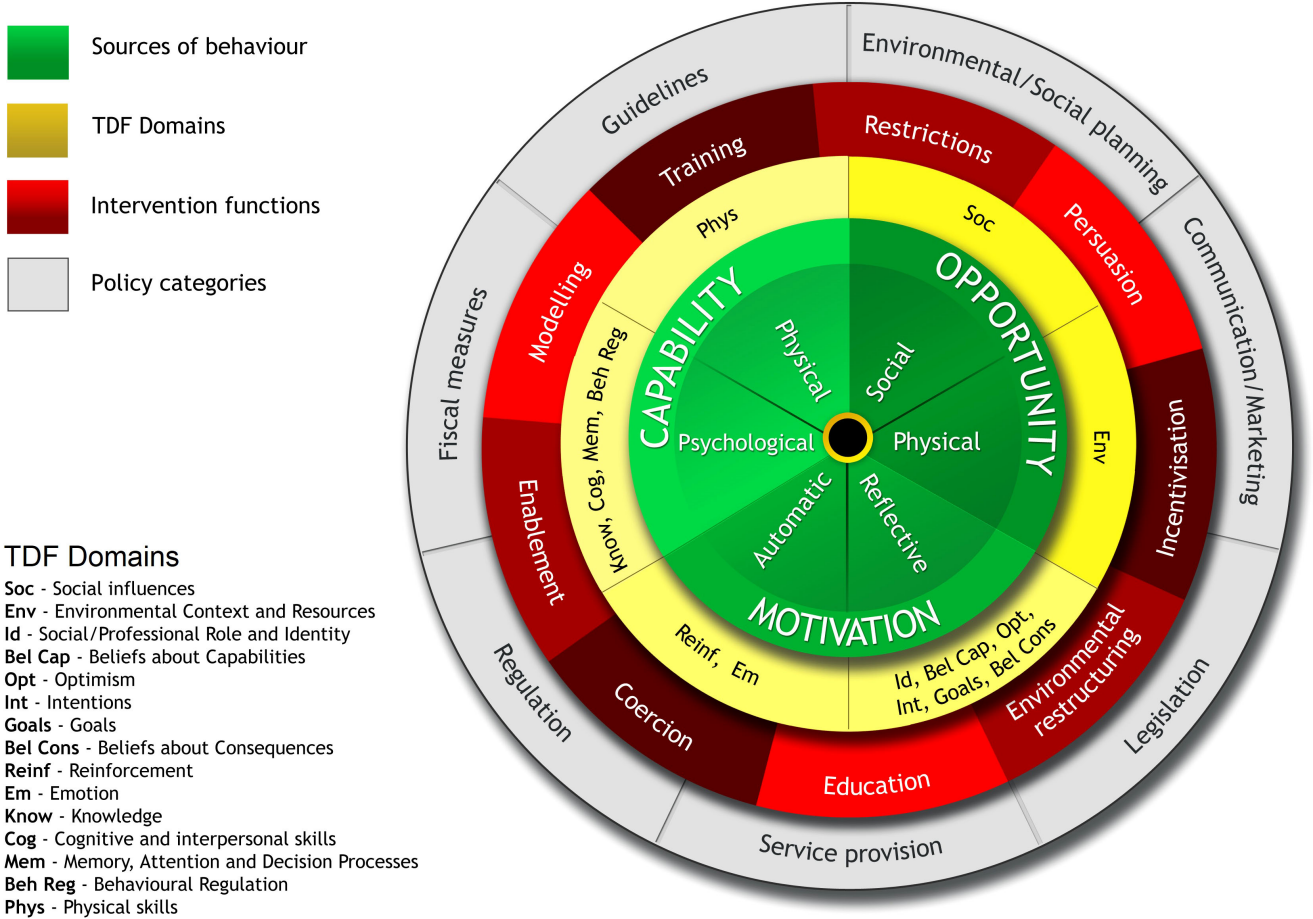
Behavior Change Wheel (reproduced with permission) (29).

The TDF maps to the multi-layered Behavior Change Wheel (BCW, **Figure 1**) (29). The BCW is an eight-step intervention development framework grounded in behavioural science. The framework has been used widely, including to address barriers to diabetes self-management (30). Adjacent to the TDF layer, and at the core of the BCW, is the Capability, Opportunity, Motivation-Behavior (COM-B) model which posits that behavior is an interaction between Capability (physical and psychological), Opportunity (physical and social) and Motivation (reflective and automatic processes). The COM-B and TDF are encircled by nine intervention functions: persuasion, incentivization, environmental restructuring, education, coercion, enablement, modelling, training, and restrictions. The nine functions and 14 TDF domains are linked to established behavior change techniques, defined as “active components of an intervention designed to change behavior” (29, 31).

While the BCW and COM-B/TDF components have demonstrated utility in identifying factors impacting health lifestyle behaviors (32, 33), to date, no studies have utilised it specifically to explore, in-depth, determinants of uptake of T2D screening among women with previous GDM. The aims of this study were to i) apply the BCW theoretical approach to identify modifiable barriers to, and enablers of, T2D screening among Australian women with prior GDM and ii) recommend intervention functions and behavior change techniques to underpin personal-level intervention messaging to optimise postpartum T2D screening uptake.

## 2 Materials and methods

### 2.1 Study design and ethics

We conducted in-depth, semi-structured interviews. Ethics approval was provided by Deakin University Human Research Ethics Committee (HEAG-H 09_2020). This study is reported according to COnsolidated criteria for REporting Qualitative research (COREQ) and Standards for Reporting Qualitative Research (SRQR, **Supplemental Materials S1-S2**) (34, 35).

### 2.2 Reflexivity

Two female researchers, both experienced in (health) psychology and qualitative research, engaged with study participants: AL is a postdoctoral research fellow, and AW is a postgraduate research assistant. They had no relationship with participants outside the study and no involvement in their clinical care. To enhance reflexivity, the interviewer (AL) completed a reflective journal with each interview, exploring perceptions, assumptions, and subjectivities.

### 2.3 Participant recruitment

All participants were registered on the National Diabetes Services Scheme (NDSS) National Gestational Diabetes Register (NGDR). The NDSS is an initiative of the Australian Government administered by Diabetes Australia. The NDSS coordinated invitations to 953 NGDR registrants via email (July 2020; reminder September). Eligibility criteria included: age 18-50 years, consent to being contacted for research purposes, 12-24 months postpartum, and English speaking. Participants registered interest via email and were telephoned by the researchers to discuss the study and schedule an interview.

### 2.4 Interview guide development

We developed a semi-structured interview guide based on the TDF domains (**Supplemental Materials S3**). The guide was pilot tested with a volunteer who had prior GDM, and minor modifications were made. In response to the COVID-19 pandemic, a question was added to invite opinions on home-based alternatives to the 6–12-week OGTT.

### 2.5 Procedure

All participants received a cover letter, plain language statement and a consent form, which they completed prior to interview (**Supplemental Materials S4**). Interviews were conducted by AL, via Zoom or telephone, from July to October 2020. Following recommendations for theory-based qualitative research, we set an *a priori* minimum sample target of N=10. At 15 interviews, we considered no new information was forthcoming (36), and conducted four additional interviews as confirmation. Each participant received an AUD$30 e-voucher and was invited to review the transcript of their interview.

### 2.6 Data handling and analysis

All interviews were audio-recorded, de-identified and transcribed professionally. Transcripts and participant details were stored digitally in password-protected files. We analysed data using NVivo (released in March 2020) (37).

#### 2.6.1 Coding framework

We developed a coding framework using an inductive-deductive model to avoid ‘rigid operationalisation’ of the TDF (38). Using an inductive approach, two researchers (AL, AW) generated theme labels for similar clusters of data. We developed theme definitions in consultation with co-authors. For the deductive element, we categorised themes into domains using a TDF-based coding manual, which contained clear statements about how the inductively generated themes would be categorised within the TDF (**Supplemental Materials S5**). We updated the coding framework and manual iteratively during data collection, practising reflexivity throughout (39).

#### 2.6.2 Data coding and analysis

After data familiarisation, two researchers (AL, AW) jointly coded one transcript. Using the manual, we coded all text relevant to the target behavior into its corresponding TDF domain, and again as a barrier or enabler. Following this, both researchers coded two (10%) transcripts independently. Inter-rater reliability was calculated using the NVivo coding comparison function (Kappa coefficient=0.73, 99% agreement). Coding differences were discussed and considered within the broader contextual meaning. Following this, one researcher (AW) coded remaining transcripts with queries addressed in consultation with co-authors. Participant responses about preferences for resource and reminders formats, and home-based alternatives to the postpartum OGTT were excluded from the main analysis but are available in **Supplemental Materials (S6).**

#### 2.6.3 Identification of ‘important’ TDF domains

We used three established criteria to identify TDF domains of ‘high importance’: frequency (total number of codes to a TDF domain), presence of conflicting beliefs/themes, and evidence of themes likely to influence behavior (39). Responses to ‘important’ TDF domains are reported below, responses to remaining TDF domains are reported in **Supplemental Materials (S7).**

#### 2.6.4 Identification of intervention functions and behavior change techniques

Using the BCW (**Figure 1**), we developed a conceptual model linking qualitative data (synthesised into the TDF domains of ‘high importance’ and mapped to COM-B elements) to intervention functions (29). We then used established procedure and taxonomies to make recommendations on behavior change techniques to underpin intervention content (29, 31).

## 3 Results

### 3.1 Participant characteristics

Twenty-one women (2%) registered interest; 19 were interviewed, two were uncontactable. Participants were (mean+SD): 34 ± 4 years, 19 ± 4 months post-partum. Most had completed OGTT within guidelines (58%), were Australian born (63%) and resided in metropolitan areas (90%), **Table 1**. Average interview duration was 41 minutes (range: 26-59). All participants reviewed and approved their transcripts; no changes were requested.

**Table 1:** Participant characteristics (N=19).

Demographic characteristic	*N*
Age (years)	34.2 ± 3.9
Education level^a^	
Year 12/VCE/Certificate/Diploma	7 (38.9)
Degree	7 (38.9)
Postgraduate	4 (22.2)
Employment status	
Home duties	5 (26.3)
Employed part-time	11 (57.9)
Employed full-time	3 (15.8)
Place of residence	
Metropolitan	17 (89.5)
Rural	2 (10.5)
Country of birth	
Australia	12 (63.2)
China	2 (10.5)
Other^b^	5 (26.5)
Language spoken at home	
English	18 (94.7)
Mandarin	1 (5.3)
Family history of type 2 diabetes	10 (52.6)
Months since pregnancy affected by gestational diabetes	18.5 ± 3.7
Uptake of screening for type 2 diabetes	
OGTT completed at some point	16 (84.2)
OGTT completed within guidelines (i.e., 6-12 weeks postpartum)	11 (57.9)
Annual screen for type 2 diabetes completed	4 (21.1)

Data are n (%) or mean ± SD; OGTT, Oral Glucose Tolerance Test; ^a^ Data missing (n=1).

^b^Other: Canada (n=1), India (n=1), Ireland (n=1), Philippines (n=1), USA (n=1).

### 3.2 Important TDF domains

Themes were coded to 13 of the 14 TDF domains. Eight domains were represented in at least two of the three importance criteria and assessed as ‘high’ importance (**Table 2**). The eight important TDF domains are described below and in **Table 3**.

**Table 2:** Identification of important TDF domains (key themes and importance criteria).

COM-B component	TDF domain	Themes	Importance criteria					Importance assessment
			Frequency^a^			Conflict^b^	Strong^c^	
			Barrier	Enabler	Total			
Capability								
Psychological	1.Knowledge	Lack of awareness: GDM-T2D link, postpartum screening	22	30	52	0	3	High
	2.Memory, attention & decision processes	Cognitive overload and memory	32	47	79	0	1	High
	3.Behavior regulation	Proactive behavior: initiating screening/ information seeking	4	19	23	0	1	Low
Physical	4.Skills	None reported	0	0	0	0	0	Low
Opportunity								
Physical	5.Environmental context & resources	Competing demands, OGTT requirements, lack of time	147	51	198	0	2	High
Social	6.Social influences	Social support (pragmatic or social/emotional); Social comparison; HCP communication about T2D risk/screening	17	106	123	0	3	High
Motivation								
Automatic	7.Reinforcement	Previous experience (OGTT, GDM management)	10	3	13	0	0	Low
	8.Emotion	Fear/anxiety related to screening or T2D diagnosis; Positive emotions (relief, reassurance, emotion (other)	26	22	48	0	2	High
Reflective	9.Beliefs about consequences	Perceived necessity of screening (beliefs about the importance and necessity of postpartum screening); Consequences of screening (beliefs about the material/ emotional consequences of screening); Anticipated outcome (screening expectations / regret)	17	86	103	1	2	High
	10.Social role/identity	Social identity (maternal role, prioritisation of own health)	11	12	23	1	1	High
	11.Beliefs about capabilities	Perceived competence (capabilities to manage own health); Perceived ability to prevent, delay or manage T2D	18	8	26	1	1	High
	12.Optimism	Perceived personal risk	4	22	26	0	1	Low
	13.Goals	Maintaining health: care for children, future pregnancy, risk management, prioritisation of financial resources	7	13	20	0	0	Low
	14.Intentions	Stated intentions to screen	2	11	13	0	0	Low
**TOTAL**			315	429	744	3	17	

^a^Frequency: number of coded responses; ^b^Conflict: number coded as conflicting beliefs; ^c^Strong: number coded as strong themes likely to affect target behavior.

**Table 3:** Eight important TDF domains with illustrative quotes.

TDF domain	Key themes	Illustrative quotes (B: barrier, E: enabler)
1.Knowledge	Knowledge and awareness screening and type 2 diabetes risk	“Diabetes is quite a silent disease until very far down the track, when you develop complications, so … what’s really going to give you a sense of what’s going on is visiting your GP and getting some blood tests” (ID01) (E) “I think there is a fair chance, realistically speaking, that I probably might end up with diabetes, but I think that’s going to be quite a few years away” (ID01) (B) “…you need to have fasted for eight hours at least, so, the easiest time to do it, I think, is in the morning” (ID08) (E)
2.Memory, attention & decision processes	Cognitive overload and memory	“…I’ve just got too much else in my life … juggling a toddler and work and house and everything” (ID08) (B) “So, I had a reminder, but I also got a letter from the hospital reminding me, and also my GP” (ID04) (E)
3.Environmental context & resources	Competing demands, OGTT requirements, time	“The doctor gave me the referral to go do it, but life was quite hectic, and I didn’t” (ID12) (B) “I think it – it is obviously hard going with a newborn” (ID10) (B) “…with work … they’re really good. I’ll say to them, ‘I need to do a test’” (ID15) (E)
	Screening requirements and environment	“It’s just very impractical … it’s not even reasonable to bring your baby to get it done” (ID03) (B) “I was just so thirsty and hungry from breastfeeding still at that time, so I found that challenging.” (ID21) (B) “…if I need to do a glucose tolerance test, I will be first priority for them [pathology laboratory]” (ID09) (E)
	Education & resources	“I also took part in a program … that was eight different online classes. They gave you information as to why this happens, what’s happening now and how to prevent it [type 2 diabetes]” (ID15) (E) “I don’t think I’ve got a reminder … I don’t know if I changed the [NDSS] detail … cause I’ve moved house (ID19). “It [information] was pretty vague.it wasn’t really focused on [screening] (ID11) (B)
4.Social influences	Communication and relationship with health professionals	“My GP was pretty good with it [screening], I actually got a text message to say, ‘You’re due to get your next glucose tolerance test.’ So, she was good at following that up” (ID08) (E) INTERVIEWER: “After pregnancy? Were you told … that there were annual tests?” INTERVIEWEE: “No. No.” (ID12) (B)
	Social support	“The thing that made it easier was knowing that I had family to look after her” (ID15) (E) “Support network is key. Maybe that could be part of the … process when people get diagnosed. What is your support network, how will you get that test post-partum, who’s going to drive you there, wo’s going to support you” (ID08) (E) “…I really struggled … just finding someone to care for my baby while I was having the test” (ID21) (B)
	Social comparison	“If I hear a story about someone else…. that could influence me, too” (ID17) (E)
5.Emotion	Fear/anxiety	“When I get the test done, it could be ‘you’re back to normal’ or it could be ‘I’m sorry, you’re already in this’”(ID06)(B) “…it does make me worry that I’m prone to it, genetically, even though there’s no one in my family with type 2 diabetes. I just need to … think about making sure that I get checked regularly and keep on top of it” (ID21) (E)
	Emotion (other)	“It was – everything was more focussed on the care of the baby.” (ID21) (B) “I’d love to see a bit more support for women post-gestational diabetes… ‘cause there was a tonne of support that I received through the hospital when I found out I had gestational diabetes.” (ID16) (B)
	Positive emotions	“… it [screening] is peace of mind for everybody involved” (ID01) (E)
6.Beliefs about consequences	Perceived necessity of screening	“…having that regular test … if I do get diabetes then I’ll know relatively early, and I think it’s more manageable the quicker you get onto it” (ID05) (E) “…you just get quite a few normal ones in a row, and you think ‘I’m all right, I’m in the clear now’” (ID01) (B)
	Consequences of screening	“…it was a confirmation that I don’t have type 2 diabetes” (ID01) (E) “…the only downsides really would be the inconvenience … and fitting that into your schedule” (ID04) (B)
	Anticipated outcome	“…try your best to get … it done, because you could get really good results, and you don’t need to worry” (ID09) (E) “…not that good, ‘cause I know my diet hasn’t been the best” (ID14) (B)
7.Social/prof. role and identity	Social identity	“…if it was for the health of my baby, I’ll do it, but if it’s just for me … it wasn’t as high on my priority list” (ID07) (B) “I feel I have to do it for, my child, and also my husband” (ID17) (E)
8.Beliefs about capabilities	Perceived competence	“…if I start to feel early symptoms, then probably that’s the time that I would go” (ID06) “I am already trying to live a healthy lifestyle that’s not too high in carbohydrate ((laughs)) - - and involves exercise, – even if I am a bit pre-diabetic, I’m actually doing mostly what I can, anyway” (ID08) (B)
	Perceived ability to manage T2D risk or T2D	“…if I were to develop type 2 diabetes (there are) things within my control to make sure that it wouldn’t be a debilitating diagnosis” (ID21) (E) “Ah, look, I guess I’m not stressed by it. I’m not anxious in any sense. Just that awareness that it could happen would help change some of my diet choices” (ID10) (E)

#### 3.2.1 Knowledge

All participants were aware of the association between GDM and T2D and understood that the purpose of postpartum screening was “…to make sure it [GDM] disappeared” (ID11). Women who understood that GDM increases the risk of developing T2D generally assigned greater importance to screening: “I’m higher risk … I have gone and done the follow-up testing” (ID10). Some noted that knowledge of the OGTT procedure and requirements acquired at GDM diagnosis was helpful as “…you know what to expect” (ID05).

Women’s knowledge of ongoing screening requirements was low: “It hadn’t registered that it was a yearly thing” (ID04) and “It’s just one blood test you have to take, is it?” (ID12). Relatedly, lack of knowledge of immediate and ongoing T2D risk translated into confusion about the rationale for regular follow-up: “I thought once I had the – the six week one, I’d be in the all-clear” (ID14).

#### 3.2.2 Memory, attention & decision processes

Women described the impact of cognitive overload associated with the demands of managing life with a baby: “I don’t think I actively decided against it [OGTT], it was just … a bit too hard” (ID01). Women also had difficulty maintaining attention to ongoing risk and screening requirements: “I put it out of my mind for a little bit … and then I forgot” (ID11). Relatedly, the asymptomatic nature of T2D meant that it was “easy to … forget when you have no symptoms” (ID01). Conversely, screening reminders were unanimously valued “my trigger to get it done” (ID03) and prompted attention to ongoing risk: “this is something you should still check-up on” (ID13).

#### 3.2.3 Environmental context & resources

**Competing demands**. Women often described how personal circumstances reduced opportunity to schedule “A time around their [newborn] chaotic routine” (ID08). This was just one of multiple competing demands the women faced: “I don’t feel like I’ve got any time ever to do things that I need to do” (ID13).

**Screening requirements and environment**. Women expressed that the requirements, timing, and duration of screening were impractical: “…you have to sit around for at least two hours, and it was just not very possible with the newborn” (ID01). For some, seeking alternate care for their newborn was undesirable: “… it’s not very practical … for a mum to leave the baby for that long” (ID03). Lack of child and nursing-friendly facilities and extended waiting times exacerbated these challenges: “…there’s just nowhere nice to sit and breastfeed and it was dusty” (ID08). Unique to the context of the COVID-19 pandemic were concerns about physical safety during the screening visit: “I don’t want to sit in a hospital around sick people for three hours … and especially now with all this Coronavirus, I definitely wouldn’t do it” (ID02).

**Education and resources**. Antenatal education was a key enabler to understanding T2D risk and importance of postpartum screening: “…in the first education session, they drilled it into us just to make sure you go” (ID04). For some, print-based resources reinforced learnings. Conversely, some noted reduction of “information after having the baby” (ID10), while others perceived that screening information was buried within an excess of “…piles of paperwork” (ID15).

#### 3.2.4 Social influences

**Social support**. Availability of practical support for the logistics of screening reduced the strain of competing demands: “I have a very supportive husband … he made sure that he was helpful with the other kids so that I could get to the appointment” (ID10). Family and friends were a powerful influence on women’s screening decisions: “… if they’re concerned about me or are trying to support me in trying to do something for my own health then I certainly respect that and listen to them” (ID16).

**Communication with health professionals**. Health professionals were regarded as a trusted and valuable source of information: “…they know your health status … if your GP calls you and tells you to do something, you generally do it” (ID14). Continuity of care reinforced the value placed on advice: “I already had that trusting relationship with them, and they knew my history … I was comfortable in her expertise in the condition” (ID14). While most women were informed about their T2D risk, few were advised about ongoing screening requirements: “She [GP] didn’t tell me” (ID02).

**Social comparison**. Knowing that other women attended screening was an enabler. For example, one participant identified closely with an online blogger who had been diagnosed with T2D after GDM, and whose narrative reinforced the importance of screening: “I had it in the back of my mind that she had developed it … I’m sure that probably had a subconscious role to play” (ID03).

#### 3.2.5 Emotion

**Fear/anxiety**. Some participants fear of T2D diagnosis impacted screening behavior: “I was worried that I would have it” (ID04). Often, this arose from perceiving T2D management as analogous to the “…regimented living style” (ID06) experienced during GDM. While fear was a clear barrier, a sense of relief and “peace of mind” (ID01) encouraged women “to keep getting the checks done” (ID21). Conversely, for a minority, an appropriate level of concern motivated screening attendance: “I was nervous that I might still have it … I wanted to get it done” (ID16). Some participants feared “needles” (ID12) or had misconceptions about potential harm: “…I felt the sugar flushed in my blood … you always worry about whether your body will react properly” (ID09).

**Postpartum abandonment**. Participants described disappointment about the rapid reduction of support after birth which greatly contrasted with experiences during pregnancy: “…the support that we received was fantastic … the minute the baby was born it, sort of, stopped” (ID16). Some described postpartum care as highly infant-focused: “I did leave that session thinking, ‘Great. My baby’s happy and healthy.’ But, for me it was, like, two questions and done” (ID04). Participants noted the need for ongoing support to prioritise their health: “reinforcing you are as important as your baby” (ID12).

#### 3.2.6 Beliefs about consequences

**Perceived necessity**. Believing that T2D screening is important for future health was a powerful enabler: “for my own body’s sake, it was the right thing to do” (ID04). Conversely, lack of symptoms and “feeling okay” (ID06) reduced perceived necessity: “I don’t feel any different from before I was pregnant, so it’s not of real urgency for me” (ID02). Perceiving GDM as transient meant screening was “less of a priority” (ID01) for some. Annual screening was sometimes perceived as unnecessary if the women had received a negative OGTT result “…it’ll be fine” (ID08) or were planning another pregnancy: “… they’ll screen me when I get pregnant again” (ID13).

**Consequences of screening**. Screening benefits typically outweighed costs. Knowing one’s health status was an enabler for some: “It’s important for women to be aware of what’s going on in their bodies, so the screening is really helpful” (ID10), but not all: “I’m not prepared to know” (ID06). For some, the appointment provided opportunity for personal time in the early postpartum period: “being able to just sit and have some time out to read a book was actually pleasant” (ID21). Most described minimal negative consequences: “It wasn’t really anything too onerous, except for a couple of hours to sit around and wait, which didn’t bother me” (ID04).

**Anticipated outcome**. For some, the possibility of undiagnosed diabetes was a motivator: “…it could be worse if I wasn’t tested regularly” (ID05). For others, anticipating that they may already have T2D was a barrier: “…it could be too late” (ID06). Expectation of a negative OGTT result was an enabler: “I knew it was going to be a normal test, so I wasn’t anxious” (ID01).

#### 3.2.7 Social professional role and identity

Social identity and motherhood role expectations were both barriers and enablers. Some women described that they “prioritise [their child’s] health over [their] own health” (ID12). For others, being a mother motivated them to screen: “…to be a good mum … you need to make sure that you’re also looking after yourself” (ID21).

#### 3.2.8 Beliefs about capabilities

**Perceived competence**. For some, compensatory strategies such as continuing to self-monitor blood glucose or engaging in healthy habits reduced the perceived necessity of screening: “…I haven’t had a test yet, but I’ve pricked myself a few times and it’s always been normal” (ID02). A minority believed that they would recognize symptoms of T2D: “I can read my body pretty well … if everything felt fine I probably wouldn’t [screen]” (ID13).

**Perceived ability to manage T2D risk**. Believing that T2D can be prevented, delayed, or managed influenced willingness to screen. High self-efficacy (i.e., belief in capability to manage risk or T2D) appeared to foster a sense of agency and reduce apprehension related to screening: “I feel like it’s within my control to a certain extent” (ID05). Conversely, one participant reported that low confidence in making diet and lifestyle change led to avoidance of screening: “I haven’t learned a different way of how to deal with stress aside from eating” (ID06).

### 3.3 Intervention functions and behavior change techniques to promote uptake of postpartum diabetes screening

**Figure 2:**
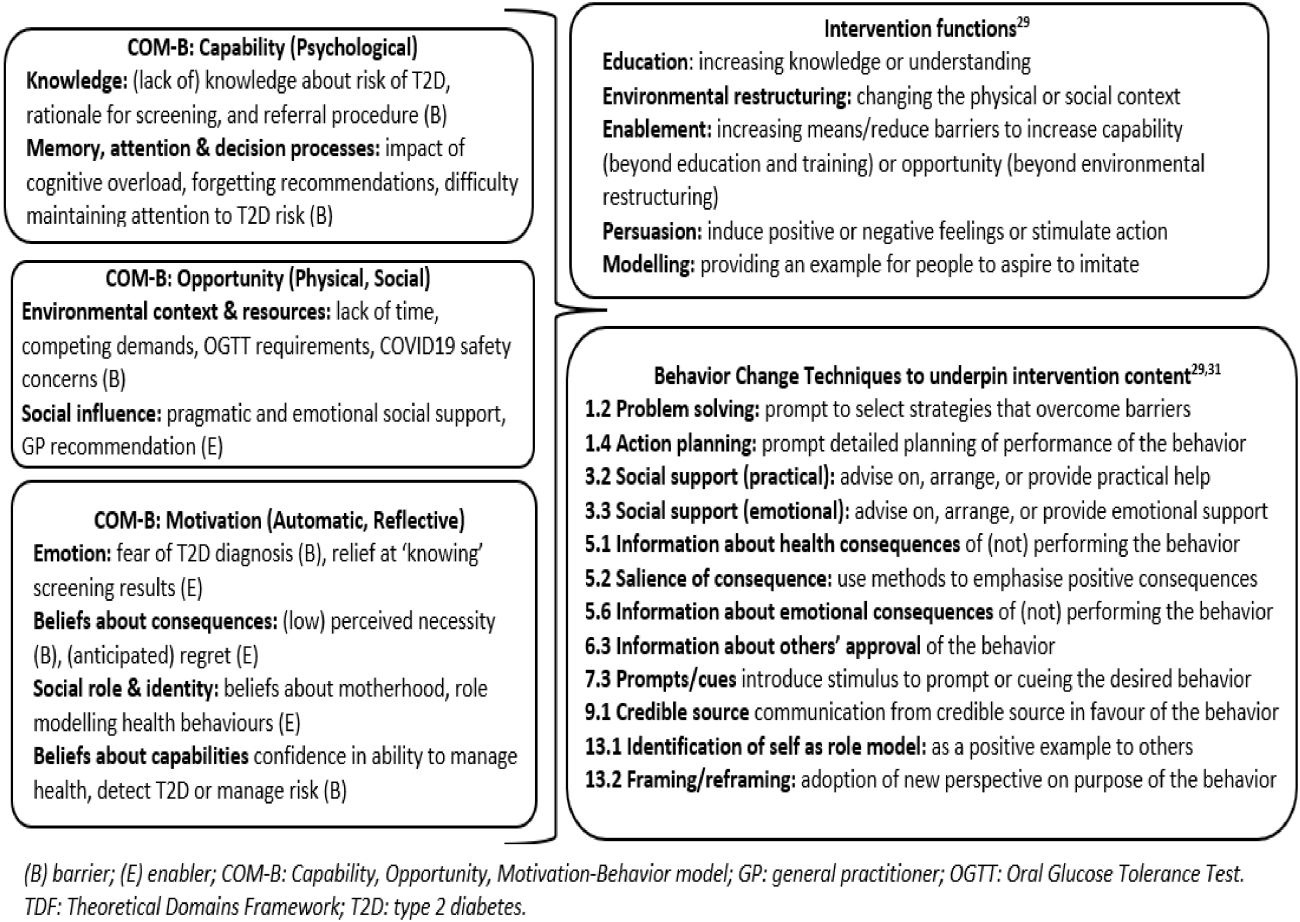
TDF domains/COM-B components linked to intervention functions and behavior change techniques.

We identified five intervention functions (education, environmental restructuring, enablement, persuasion, modelling) and 12 behavior change techniques to address the eight ‘important’ TDF domains (29). We illustrate the linkages from evidence (synthesised into TDF domains) to intervention components (functions and behavior change techniques) in **Figure 2**.

## 4 Discussion

Using a methodology grounded in behavior change theory, we identified multiple modifiable determinants of T2D screening uptake among Australian women with prior GDM. Eight ‘high’ importance TDF domains, mapped onto the COM-B model, provide an evidence base for future messaging development and intervention targets.

Participants had high awareness of T2D risk and the rationale for screening, consistent with prior Australian studies (16, 18, 19, 21). Linkages between TDF domains provide context for this finding. Knowledge was acquired via health professional advice (‘social influences’) and education/resources (‘environmental context & resources’). Despite this knowledge, some participants reported a missed or delayed OGTT, emphasising the need for messaging that targets additional psychosocial factors.

Knowledge related to ongoing T2D screening and risk appears to be lacking, as evidenced by confusion about frequency, test type and rationale for periodic screening. This was likely influenced by the temporary suspension of NGDR annual reminders in response to changes to guidelines (40) for postpartum testing during the COVID-19 pandemic. However, uptake in Australia is typically low (6). Our findings suggest that this reflects cognitive overload due to fatigue and parenting demands, and a disproportionate emphasis on the onerous 6-12-week OGTT, whereas future follow-up is the simpler fasting glucose or HbA1c (4, 12–14). Two distinguishing screening enablers were maternal identity and social-emotional support. These novel findings suggest that addressing stereotypical norms and beliefs about maternal role and providing emotional support may be effective in motivating screening uptake.

### 4.1 Comparison with previous research

Previous research has recommended the use of system-based or local reminders, as forgetting is a common barrier to screening (12). This was not identified in the present study. While our findings contrast with prior Australian qualitative research (15, 18), they may reflect system-based improvements in the NGDR and high registration rate (6). Prior research has indicated that NGDR reminders are not effective in promoting screening uptake (6, 22). Our research confirms that women value reminders and there is scope to incorporate messages targeting the psychosocial barriers and enablers identified in this study.

Competing demands and screening-related challenges were key personal-level barriers in this and other studies (14). Prior recommendations suggest altering the physical environment to be more ‘baby friendly’ (12). Women’s expressed concerns regarding physical safety in the context of the COVID-19 pandemic challenge this recommendation; supported by findings from a recent Danish study (41). Instead, clinician support and encouraging women to seek practical social support (a key enabler in overcoming situational and contextual barriers) may be more pragmatic (26).

Concordant with prior research, women often prioritised their child’s needs over their own health (14). Reduced healthcare information and support postpartum (‘postpartum abandonment’) was disempowering and served to confirm women’s perceptions of their own health being less important, an issue identified in a recent scoping review (26).

Fear of T2D diagnosis was the most frequently reported motivational barrier, and often related to extrapolation of prior experience of GDM management to future diabetes management. Fear is a widely identified emotion in health behavior change research, including for postpartum diabetes screening (14). While appropriate concern is a necessary component of risk perception, fear is unlikely to promote behavior change, particularly in the absence of self-efficacy and response efficacy (i.e., a person’s belief that changing their behavior will reduce risk) (42). The present findings align with this behavioral pathway. For example, key enablers were understanding personal risk and perceiving screening as an important health behavior that may mitigate potential adverse health consequences (high response efficacy). Conversely, barriers included low, or distal representations of personal risk and lack of perceived benefit, or necessity, of screening (low response efficacy). Similar findings, reported elsewhere, suggest that addressing beliefs and perceived personal risk is essential in increasing T2D screening uptake (12–14).

Relatedly, a major screening barrier for some women was perceived competence in managing their own health, mostly among those who had delayed or not attended screening. Misplaced beliefs in their own capability to recognize T2D symptoms reduced the priority women assigned to formal screening (14). Previous research noted use of compensatory strategies (e.g. self-testing) in-lieu of formal screening (14). Finally, while home-based alternatives to formal OGTT may be attractive and viable options in future (43), further research is needed to establish their feasibility and effectiveness, given the concerns raised by the women in this study. Collectively, these findings indicate the need for education to address misconceptions about the ability to recognize T2D symptoms, and the rationale for timely, formal screening and ongoing surveillance.

These findings suggest the potentially important role of self-efficacy in screening uptake. Low self-efficacy, regarding personal capability to prevent or manage T2D, negatively impacted willingness to screen, which has been noted in only two other Australian studies (15, 21). Uniquely, our study also shows the positive influence of self-efficacy, which has not been previously reported. This novel finding aligns with research proposing the mediating relationship between fear and self-efficacy and suggests the potential value of increasing self-efficacy through empowerment-based messaging to increase screening uptake (42).

### 4.2 Implications for policy and practice

#### 4.2.1 Policy

Prior recommendations to increase screening uptake typically targeted knowledge (12, 13). While an important prerequisite, our findings suggest that ‘Knowledge’ is already broadly addressed by existing educational policies in an Australian context. However, screening prompts and reminders provided by Australia’s National Gestational Diabetes Register could be improved by review to ensure that the messaging both addresses the determinants identified in this study and is underpinned by the recommended behaviour change techniques. Most of the modifiable determinants of T2D screening uptake relate to the Opportunity and Motivation elements of the COM-B model. Therefore, screening uptake is most likely to be improved by timely (44), person-centred messaging that addresses opportunistic barriers (e.g., competing demands and elements of the screening procedure), motivational factors (e.g., emotions, beliefs, self-efficacy, maternal identity).

A further consideration for policymakers is the current reliance on the 6–12-week OGTT, which is a barrier to screening and could be replaced with a simpler fasting glucose, HbA1c or home-based alternatives. This would require further research to establish their feasibility and effectiveness. Finally, the women’s concerns about postpartum abandonment suggest that greater consideration needs to be given to ensuring appropriate handovers from tertiary to primary care, and continued support during this time.

#### 4.2.2 Clinical practice

We have identified behavior change techniques to underpin psychoeducational messaging (**Figure 2**). These techniques can be extrapolated for use in the clinical context. For example, clinicians could promote screening uptake through providing accurate and timely information about T2D risk, the rationale and positive health consequences of screening. It is important that clinicians use a gain-framed approach to emphasize the potential positive outcomes of screening such as reassurance if screening is negative and the ability to access timely and effective treatments if screening is positive. Clinicians are well placed to arrange or address practical or emotional support to overcome the unique barriers that the woman may be experiencing. Further, clinicians can frame screening as an important role modelling behavior which is of direct benefit to the health and wellbeing of the family (44). Specific communication points for clinicians are reported in a recent systematic review published by our research team (14).

### 4.3 Strengths and limitations

This study was limited by the homogenous sample: most participants reported having attended the postpartum OGTT (some overdue). Participation rate was low, and recruitment coincided with the onset of the COVID-19 pandemic, suggesting that those who participated were highly engaged. All participants were fluent English speakers, and most lived in metropolitan areas and had strong social networks. Further, the generalizability to Indigenous women and culturally and linguistically diverse populations is limited by the predominantly Anglo-Australian participants. Recognising the inherent limitations of representativeness (45), our findings provide valuable insight into enabling factors and strategies to overcome screening barriers.

A key strength is the study design, underscored by explicit use of theoretical approach and intervention development frameworks (25). Data collection was optimised by piloting the interview schedule and including follow-up prompts to promote in-depth exploration (39). Several strategies were implemented to enhance the trustworthiness of the data, including participant checking, reflective journals, audit trails and involvement of multiple researchers at each stage of the analysis (34, 35).

### 4.4 Future research

Next steps involve developing discrete, person-centered psycho-educational messages for implementation within the national register and other delivery approaches. We have proposed behavior change techniques to underpin the messaging (**Figure 2**) although others shown to be associated with significant improvements in diabetes self-management (e.g., goal setting) (46) should also be considered.

Finally, further research is needed to establish the unique behavioral determinants of screening among women from culturally diverse and minority backgrounds. Indigenous, Chinese, and non-English speaking women are at greater risk of T2D than women of White European ethnicity. Given that the former experience unique barriers to T2D prevention and screening (3, 47, 48) this needs to be a priority area for future research (48).

### 4.5 Conclusions

This study identified numerous modifiable barriers and enablers to postpartum T2D screening for women with prior GDM. By mapping to the BCW, we identified intervention functions and behavior change techniques to underpin intervention content. These findings provide a valuable evidence base for developing messaging and interventions that target the behavioral determinants most likely to optimise T2D screening uptake among women with prior GDM. 

## Resource identification initiative

NVivo (RRID : SCR_014802).

## Data availability statement

Additional detail about the datasets analyzed for this study can be found in supplemental materials. The data generated and analyzed during the current study are not publicly available due to restrictions on sharing qualitative data. Requests to access the datasets should be directed to Dr Amelia J Lake (E: alake@acbrd.org.au).

## Ethics statement

The studies involving human participants were reviewed and approved by Deakin University Human Research Ethics Committee (HEAG-H 09_2020). The participants provided their written informed consent to take part in this study.

## Author contributions

AL designed the interview guide and TDF-based coding manual in consultation from co-authors. AL conducted all interviews. AL and AW coded, analysed and interpreted data and prepared the manuscript with input, review, and approval from all co-authors. All authors contributed to the article and approved the submitted version.
