# Supplementary material for: Barriers to and enablers of type 2 diabetes screening among women with prior gestational diabetes: A qualitative study applying the Theoretical Domains Framework
Source: Front Clin Diabetes Healthc. 2023 Feb 24;4:1086186. doi: 10.3389/fcdhc.2023.1086186 (PMC10012118; doi:10.3389/fcdhc.2023.1086186)
Supplement: Supplementary file 1 [file DataSheet_1.docx]

Supplementary Material

**Supplemental material overview**

| **Number** | **Description** | **Page number** |
| --- | --- | --- |
| S1 | COREQ checklist | 2 – 3 |
| S2 | SRQR checklist | 4 - 5 |
| S3 | TDF-based interview guide | 6 - 10 |
| S4 | Cover letter, plain language statement and consent forms | 11 - 16 |
| S5 | TDF-based coding manual | 17 – 28 |
| S6 | Participant preferences for resources and reminders, and alternatives to the 6-12-week OGTT | 29 – 31 |
| S7 | TDF domains of ‘less’ importance | 32 – 34 |

# S1 Table 1: COnsolidated criteria for REporting Qualitative research (COREQ) checklist

| **Topic** | **Item No.** | **Guide questions/description** | **Manuscript**  **page no.** |
| --- | --- | --- | --- |
| **Domain 1. Research team and reflexivity** | | | |
| *Personal characteristics* | | | |
| Interviewer/facilitator | 1 | Which author/s conducted the interview or focus group? | P4 |
| Credentials | 2 | What were the researcher’s credentials? E.g., PhD, MD | P3 |
| Occupation | 3 | What was their occupation at the time of the study? | P3 |
| Gender | 4 | Was the researcher male or female? | P3 |
| Experience and training | 5 | What experience or training did the researcher have? | P3 |
| *Relationship with participants* | | | |
| Relationship established | 6 | Was a relationship established prior to study commencement? | P4 |
| Participant knowledge of the interviewer | 7 | What did the participants know about the researcher? e.g., personal goals, reasons for doing the research | S4 |
| Interviewer characteristics | 8 | What characteristics were reported about the interviewer/facilitator? e.g., Bias, assumptions, reasons, and interests in the research topic | P3-4 |
| **Domain 2: Study design** | | | |
| *Theoretical framework* | | | |
| Methodological orientation and Theory | 9 | What methodological orientation was stated to underpin the study? E.g., grounded theory, discourse analysis, ethnography, phenomenology, content analysis | P4; F1, S3 |
| *Participant selection* | | | |
| Sampling | 10 | How were participants selected? E.g., purposive, convenience, consecutive, snowball | P4 |
| Method of approach | 11 | How were participants approached? e.g., face-to-face, telephone, mail, email | P4 |
| Sample size | 12 | How many participants were in the study? | P5 |
| Non-participation | 13 | How many people refused to participate or dropped out? Reasons? | P5 |
| *Setting* | | | |
| Setting of data collection | 14 | Where was the data collected? e.g., home, clinic, workplace | P4 |
| Presence of non-participants | 15 | Was anyone else present besides the participants and researchers? | P4 |
| Description of sample | 16 | What are the important characteristics of the sample? e.g., demographic data, date | P5, T1 |
| *Data collection* | | | |
| Interview guide | 17 | Were questions, prompts, guides provided by the authors? Was it pilot tested? | P5, S5 |
| Repeat interviews | 18 | Were repeat inter views carried out? If yes, how many? | N/A |
| Audio/visual recording | 19 | Did the research use audio or visual recording to collect the data? | P4 |
| Field notes | 20 | Were field notes made during and/or after the interview or focus group? | P4 |
| Duration | 21 | What was the duration of the interviews or focus group? | P5 |
| Data saturation | 22 | Was data saturation discussed? | P4 |
| Transcripts returned | 23 | Were transcripts returned to participants for comment and/or correction | P5 |
| **Domain 3: analysis and findings** | | | |
| *Data analysis* | | | |
| Number of data coders | 24 | How many data coders coded the data? | P5 |
| Description of coding tree | 25 | Did authors provide a description of the coding tree? | P5, S5 |
| Derivation of themes | 26 | Were themes identified in advance or derived from the data? | P4 |
| Software | 27 | What software, if applicable, was used to manage the data? | P4 |
| Participant checking | 28 | Did participants provide feedback on the findings? | No |
| *Reporting* | | | |
| Quotations presented | 29 | Were participant quotations presented to illustrate the themes/findings? Was each quotation identified? e.g., participant number | P6-8, T3, S6 |
| Data and findings consistent | 30 | Was there consistency between the data presented and the findings? | P6-8, T2, T3, F2 |
| Clarity of major themes | 31 | Were major themes clearly presented in the findings? | T2, F2 |
| Clarity of minor themes | 32 | Is there a description of diverse cases or discussion of minor themes? | P6-8, P9-10, S6 |

F: Figure number, P: page number(s), S: supplementary materials section; T: in-text table number

# S2 Table 2: Standards for Reporting Qualitative Research (SRQR) checklist

| **No.** | **Item** | **Description** | **Page** |
| --- | --- | --- | --- |
| **Title and abstract** | | | |
| S1 | Title | Concise description of the nature and topic of the study Identifying the study as qualitative or indicating the approach (e.g., ethnography, grounded theory) or data collection methods (e.g., interview, focus group) is recommended | P1 |
| S2 | Abstract | Summary of key elements of the study using the abstract format of the intended publication; typically includes background, purpose, methods, results, and conclusions | P1-2 |
| **Introduction** | | | |
| S3 | Problem formulation | Description and significance of the problem/phenomenon studied; review of relevant theory and empirical work; problem statement | P2-3 |
| S4 | Purpose of research question | Purpose of the study and specific objectives or questions | P3 |
| **Methods** | | | |
| S5 | Qualitative approach and research paradigm | Qualitative approach (e.g., ethnography, grounded theory, case study, phenomenology, narrative research) and guiding theory if appropriate; identifying the research paradigm (e.g., postpositivist, constructivist/ interpretivist) is also recommended; rationale** | P4-5, F1, S4 |
| S6 | Researcher characteristics and reflexivity | Researchers’ characteristics that may influence the research, including personal attributes, qualifications/experience, relationship with participants, assumptions, and/or presuppositions; potential or actual interaction between researchers’ characteristics and the research questions, approach, methods, results, and/or transferability | P3-4 |
| S7 | Context | Setting/site and salient contextual factors; rationale** | P4 |
| S8 | Sampling strategy | How and why research participants, documents, or events were selected; criteria for deciding when no further sampling was necessary (e.g., sampling saturation); rationale** | P4 |
| S9 | Ethical issues pertaining to human subjects | Documentation of approval by an appropriate ethics review board and participant consent, or explanation for lack thereof; other confidentiality and data security issues | P3, S4 |
| S10 | Data collection methods | Types of data collected; details of data collection procedures including (as appropriate) start and stop dates of data collection and analysis, iterative process, triangulation of sources/methods, and modification of procedures in response to evolving study findings; rationale** | P4 |
| S11 | Data collection instruments and technologies | Description of instruments (e.g., interview guides, questionnaires) and devices (e.g., audio recorders) used for data collection, if/how the instrument(s) changed over the course of the study | P4, S3 |
| S12 | Units of study | Number and relevant characteristics of participants, documents, or events included in the study; level of participation (could be reported in results) | P5, T1 |
| S13 | Data processing | Methods for processing data prior to and during analysis, including transcription, data entry, data management and security, verification of data integrity, data coding, and anonymization/de-identification of excerpts | P4 |
| S14 | Data analysis | Process by which inferences, themes, etc., were identified and developed, including the researchers involved in data analysis; usually references a specific paradigm or approach; rationale** | P4-5, S5 |
| S15 | Techniques to enhance trustworthiness | Techniques to enhance trustworthiness and credibility of data analysis (e.g., member checking, audit trail, triangulation); rationale** | P4 |
| **Results/findings** | | | |
| S16 | Synthesis and interpretation | Main findings (e.g., interpretations, inferences, and themes); might include development of a theory or model, or integration with prior research or theory | P5-8, F2, T2, T3 |
| S17 | Links to empirical data | Evidence (e.g., quotes, field notes, text excerpts, photographs) to substantiate analytic findings | P5-8, F2, T2, T3, S6, S7 |
| **Discussion** | | | |
| S18 | Integration with prior work, implications, transferability, and contribution(s) to the field | Short summary of main findings; explanation of how findings and conclusions connect to, support, elaborate on, or challenge conclusions of earlier scholarship; discussion of scope of application/generalizability; identification of unique contribution(s) to scholarship in a discipline or field | P9-10 |
| S19 | Limitations | Trustworthiness and limitations of findings | P11 |
| **Other** | | | |
| S20 | Conflicts of interest | Potential sources of influence or perceived influence on study conduct and conclusions; how these were managed | P12 |
| S21 | Funding | Sources of funding and other support; role of funders in data collection, interpretation, and reporting | P12 |

F: Figure number, P: page number (starting page in manuscript), S: supplementary materials section; T: in-text table number

# S3 TDF-based interview guide

*You have been invited to take part in this study because you have previously had gestational diabetes. The interview should take around 30-45 minutes. We can stop at any time and start again later if you need to. I will record our conversation but before I do, I want to emphasise that what we discuss is confidential and will not affect your relationship with your healthcare professionals or the National Diabetes Services Scheme.*

*When we finish, the recording will be given an anonymous identifier number and the only link between it, and you will be kept in a secure file which can only be accessed by me, and the other researchers listed in the Plain Language Statement. You can skip any questions that you want, and you can withdraw from the study at any time.*

**Demographic questions** *I would like to start by asking some questions about you so that we understand who took part in our study. Your responses will be de-identified and will only be described as part of the group who participated.*

1. What is your marital status? (e.g., married/partnered, not partnered)
2. Does anyone in your family have type 2 diabetes?

- If yes, what is your relationship to them? (e.g., is the person your parent, sibling?)

1. What is your employment status? (Employed, Unemployed, Student, home duties)

If employed, what is your usual occupation?

*Now I will ask a couple of questions about your experience of gestational diabetes*

1. How many months since your pregnancy with gestational diabetes?
2. What was your experience of having gestational diabetes?

- *(Prompt: what were you told? How did you manage the condition?)*

1. Could you tell us in your own words what gestational diabetes is?

- *(Prompt: factors that put a woman at risk of GDM? Any short- or long-term impacts of GDM?)*

1. Has having gestational diabetes impacted your life since?
2. Have you had other pregnancies where you had gestational diabetes?
3. Have you been diagnosed with type 2 diabetes since your pregnancy?

**Type 2 diabetes.** *Gestational diabetes usually goes away immediately after pregnancy. Women who have had gestational diabetes are at risk of developing type 2 diabetes later.*

1. Before taking part in this study, were you aware of a connection between gestational diabetes and type 2 diabetes? If yes, how did you become aware?
2. Can you tell me in your own words what you understand about the connection between gestational diabetes and type 2 diabetes?
3. Was the link discussed with you when you were first diagnosed or during pregnancy?

- *(Prompt: What was said? How did that make you feel?)*

1. What do you know about type 2 diabetes?
2. Do you think you are likely to develop type 2 diabetes in the future?
3. Does risk of developing Type 2 diabetes concern you? (Prompt: can you tell me why?)
4. Do you know of anything that a woman can do to reduce her risk of getting type 2 diabetes after pregnancy? If yes, prompt for detail.

**Screening for type 2 diabetes (the OGTT**). *An oral glucose tolerance test or OGTT is used to screen for gestational diabetes during pregnancy. The OGTT involves fasting overnight, drinking a sweet solution, waiting ~2 hours, then providing a blood sample. Do you remember taking the test? Most women who have had gestational diabetes are invited to take a screening test for type 2 diabetes after pregnancy.*

1. Do you know when a woman should screen for type 2 diabetes after pregnancy?

- If yes, prompt for further detail

*Most women who have had gestational diabetes receive a reminder letter (or invitation) to have an OGTT screening test 6-12 weeks after pregnancy and then yearly blood tests. Some women do not take the screening tests and there are many reasons for this.*

1. Did you receive a reminder letter to have the OGTT at around 6-12 weeks after pregnancy?

*Most of the questions from here are designed to get an understanding of what makes it harder or easier to take part in the type 2 diabetes screening tests. Some may sound repetitive, but they help to best understand your point of view.*

1. Did you have the first OGTT after your pregnancy?

- If yes, thinking back to that time…
  1. Was it in the recommended time frame (i.e., 6-12 weeks after pregnancy)?
  2. Did you have to remind yourself to go?
  3. What things made it hard for you to take the screening test?
  4. What things made it easier?
  5. What feelings did you have before the OGTT?
  6. What feelings did you have during the OGTT?
  7. What feelings did you have when you received the results?
- If no, thinking back to that time…can you tell me why not?

1. Did you consciously decide against the OGTT?
2. Please step me through how you made the decision not to have the OGTT?
3. What things made it hard for you to take the screening test?
4. What could have made it easier?
5. Considering your life in the first 3 months after pregnancy, how important was it for you to screen for type 2 diabetes then? *(Prompt: what were higher priorities and why?)*
6. How would you explain the OGTT to other women?
   1. procedurally, and
   2. emotionally, to encourage the woman to go
7. Hypothetically, if you DID need to take the OGTT again in the future, what would help you to undertake the test?
8. Please describe how to go about taking the test? (*Prompt: steps in detail)*

**COVID-19 question (exploring alternatives to in-person pathology attendance).**

1. What do you think about the option of replacing the OGTT with a fasting blood glucose from home, using your own glucose meter?
2. How would you feel about taking a photo of the glucose meter reading and sending it to your GP?
3. Would you prefer a reminder to test?
4. Would you prefer to finger prick or use a glucose meter?

**Screening for type 2 diabetes (annual HbA1C).** *All women registered with the National Diabetes Services Scheme receive reminder letters to have annual type 2 diabetes tests, staring a year after their pregnancy. Your GP will advise you on the type of test, but this usually involves taking blood to check for blood glucose levels. Irrespective of whether you took the test…*

1. Did you receive a reminder letter from the NDSS?

If yes, what thoughts and feelings did you have?

1. Did you have the annual type 2 diabetes screen blood test?

If yes, thinking back to that time…

1. Was it in the recommended time frame (i.e., one year after pregnancy)? Y/N
2. Did you have to remind yourself to go?
3. What things made it hard for you to take the screening test?
4. What things made it easier?
5. What feelings did you have before the blood test?
6. What feelings did you have during the blood test?
7. What feelings did you have you received the results?

If no, thinking back to that time…

1. Can you tell me why not?
2. Did you consciously decide not to take the test? *(Prompt: what were the reasons for the decision)?*
3. What things made it hard for you to take the screening test?
4. What could have made it easier?
5. Considering your life now, how important is it for you to screen for type 2 diabetes?

*(Prompt: What are higher priorities and why?)*

1. How would you explain the annual screening blood test to other women?
2. procedurally, and
3. emotionally, to encourage the woman to go

**Screening for type 2 diabetes (overall).**

*All NDSS registrants will continue to receive annual reminders to screen for type 2 diabetes*

1. What do you think about that?
2. How do you feel about that?
3. What do you believe are the benefits to the screening tests?
4. Are there are any negatives or ‘down sides’ to the screening tests?

*(Prompt: Does one outweigh the other?)*

**Social influence factors, gestational diabetes, and screening.**

1. How comfortable were you with people knowing that you had gestational diabetes?

*(Prompt: why is that?)*

1. (Since birth) have your views changed, or stayed the same?
2. How comfortable are you with people knowing that you are being asked for screen for type 2 diabetes on a regular basis? *(Prompt for detail)*
3. Have you been prompted by anyone to screen for type 2 diabetes after pregnancy?

- If yes, who?

1. If anyone could influence your decision to screen for type 2 diabetes, who would it be?

- *(Prompt: family, friends, support group, health professional, community leader, celebrity)*
- (If some listed) why would their views influence you?

1. Do you know of anyone who has health issues because of diabetes?

- If yes, did it influence your choice to attend screening for T2D?

1. Has anyone you know been tested for type 2 diabetes after pregnancy?

- *(Prompt for detail, emotional response).*

**Beliefs about capabilities.**

1. How confident are you that you can talk about gestational diabetes with your GP?

- *(Prompt: what makes it easy/hard?)*

1. What do you think would help you to overcome these problems?
2. How confident are you that you can talk about screening for type 2 diabetes with your GP? *(Prompt: what makes it easy/hard?)*
3. What do you think would help you to overcome these problems?

*We’re almost done, but before I wrap it up, I would like to ask you about your preferences for information about screening for type 2 diabetes.*

**Communication materials.**

1. How/where do you get information about looking after your health after gestational diabetes?
2. What information have you found helpful? *(Prompt: source AND format)*
3. What information or messaging would you like to see, to promote uptake of the OGTT?
4. How would you like to be reminded to go for the initial OGTT and the annual screening from now on? *(Prompts: postal reminder, SMS, e-mail, app?)*
5. What other improvements would you suggest? *(Prompts: messaging and materials)*

**Closing, invitation to review transcript and messaging**

*Thank you very much for taking part in this interview.*

1. Is there anything else about this topic that you would like to mention?

*(Prompt: I’m particularly interested in the testing that you have been asked to take for type 2 diabetes).*

Would you like to review a typed transcript of our discussion to confirm that your views have been recorded properly? If yes: I will email you when it is ready for review.

We are going to use the information from the interviews to develop new resources, which promote type 2 diabetes screening for women after gestational diabetes.

Would you like to review the materials? If yes, I will email you when it is ready for review.

**Risk protocol.**

If the participant expresses distress, consider advising support seeking from:

- family member or friend,
- primary maternity care provider (e.g., GP or maternal and child health nurse),
- peer support groups.

The following free services are also available:

- If participant has experienced a pregnancy loss due to miscarriage, stillbirth, or neonatal death: Miscarriage, Stillbirth & Neonatal Death support network (SANDS) on 13 000 SANDS (13 000 72637).
- Beyond Blue 1300 22 4636, http://www.beyondblue.org.au/ (web chat every day from 3:00 PM until 12:00 AM (AEDST)
- Lifeline 131146

**FAQs and credible sources.**

https://www.ndss.com.au/about-diabetes/pregnancy/gestational-diabetes/

https://www.diabetesaustralia.com.au/gestational-diabetes

**Benefits of 6–12-week OGTT.** The major benefit of the 6–12-week OGTT is early detection of type 2 diabetes. If high blood glucose levels (prediabetes) or type 2 diabetes are detection, management and treatment strategies can be put in place to slow progression of the condition.

**Risk factors for GDM.** Previous gestational diabetes; age; ethnicity (Aboriginal and Torres Strait Islander, Melanesian, Polynesian, Indian subcontinent, Middle Eastern or Asian background); elevated blood glucose levels in the past; family history of type 2 diabetes or first-degree relative (mother or sister) who has had gestational diabetes; above healthy weight range; polycystic ovary syndrome; rapid weight gain in first half of pregnancy; have had a large baby (weighing more than 4,500g) or complications in a previous pregnancy; or are taking some types of antipsychotic or steroid medications

**Managing GDM:** refer ‘gestational diabetes: caring for yourself and baby’ booklet, sent to NGDR registrants when first diagnosed and registered with the NGDR, ~7 months gestation). Main activities: healthy eating plan, regular physical activity, monitoring blood glucose levels, and taking medication (if needed).

# S4 Cover letter, plain language statement and consent forms

# S5 Table 3: TDF-based coding manual

| TDF domain and definition | | Component constructs | Example coding guidelines applied to target behaviour |
| --- | --- | --- | --- |
| 1. Knowledge   Awareness of the existence of something | Knowledge (including knowledge of condition / scientific rationale)  Procedural knowledge: Knowing how to do something  Knowledge of task environment: the social and material context in which a task is undertaken | | Knowledge may be both correct and incorrect but must relate/link to attending T2D screening. In the context of type 2 diabetes (T2D) screening for women with previous gestational diabetes (GDM), knowledge could relate to discussion about:   - antecedents to GDM and T2D - gestational diabetes, T2D (including severity), and the link between the two (clinical, prevalence, risk of conversion) - need or rationale for T2D screening post GDM - screening procedure for T2D screening tests (e.g., time points or frequency, preparatory behaviours, screening process) *except* when discussion is on how easy/difficult it is to attend screening (*beliefs about capabilities*) - outcome management *except* when comment has emotional element (*emotion*)   Alternate coding for related material (depending on the context):   - Information from health professionals where an individual’s thoughts, feelings of behaviours have changed as a result (*social influences*) - Resources or learnings which have encouraged the development of skills etc *(environmental context and resources*) - Discussion of ease/difficulty in attending T2D screening (beliefs about capabilities OR environmental context and resources OR beliefs about capabilities – if confidence is an issue) |
| 1. Skills   Ability or proficiency acquired through practice | | Skills: Ability or proficiency acquired through practice  Skills development: the gradual acquisition or advancement through progressive stages of an ability or proficiency acquired through training and practice  Competence: skills, and ability as it is applied to a task or set of tasks  Ability: Competence or capacity to perform a physical or mental act. Ability may be either unlearned or acquired by education and practice  Interpersonal skills: An aptitude enabling a person to carry on effective relationships with others, such as an ability to cooperate, to assume appropriate social responsibilities or to exhibit adequate flexibility  Practice: Repetition of an act, behaviour, or series of activities, often to improve performance or acquire a skill  Skill assessment: A judgement of the quality, worth, importance. Level or value of an ability or proficiency acquired through training and practice | Skills may be both present and absent. In the context of type 2 diabetes (T2D) screening for women with previous gestational diabetes (GDM), skills could relate to discussion about:   - performance of home-based blood glucose testing (as an alternative to the OGTT), including working the glucose meter correctly, documenting results, sending to healthcare professional.   Sometimes double coded *Beliefs about capabilities* (e.g., when discussing ability to communicate about T2D screening in English as a second language)  Alternate coding for related material (depending on the context):   - Antenatal/postnatal education and whether it improved uptake of T2D screening (*environmental context and resources -* availability of education and the influence on ability for screening). |
| 1. Social professional role and identity   A coherent set of behaviours and displayed personal qualities of an individual in a social or work setting | | Professional identity: the characteristics by which an individual is recognised relating to, connected with, or befitting a particular profession  Professional role: the behaviour considered appropriate for a particular kind of work or social position  Social identity: the set of behavioural or personal characteristics by which an individual is recognizable [and portrays] as a member of a social group  Identity: an individual’s sense of self defined by a) a set of physical and psychological characteristics that is not wholly shared with any other person and b) a range of social and interpersonal affiliations (e.g., ethnicity) and social roles  Professional boundaries: the bounds or limits relating to or connected with a particular profession or calling.  Professional confidence: an individual’s belief in his or her repertoire of skills and ability especially as it is applied to a task or set of tasks.  Group identity: the set of behavioural or personal characteristics by which an individual is recognizable [and portrays] as a member of a group.  Leadership: the processes involved in leading others, including organising, directing, coordinating, and motivating their efforts toward achievement of certain group or organisation goals  Organisational commitment: An employee’s dedication to an organisation and wish to remain part of it. Organisational commitment is often described as having both an emotional or moral element and a more prudent element. | In the context of type 2 diabetes (T2D) screening for women with previous gestational diabetes (GDM), identity could relate to discussion about:   - social identity as a woman diagnosed with gestational diabetes - identity of being a mother (e.g., prioritising care for baby over care for self-including T2D screening) - view of a typical person who attends/does not attend T2D screening - how cultural background influenced T2D screening attendance (including discussion of how culture affects screening behaviour of others in participants’ culture) - illness identity (e.g., perception of stigma around GDM and screening for T2D)   Alternate coding for related material (depending on the context):   - Descriptions of relationship influences (*social influences*)   Note: In this circumstance social and personal identity constructs apply. Professional role and identity are less relevant. |
| 1. Beliefs about capabilities   Acceptance of the truth/reality about or validity of an ability, talent, or facility that a person can put to constructive use. | | Self-confidence: Self-assurance or trust in one’s own abilities, capabilities, and judgement  Perceived competence: An individual’s belief in her or her ability to learn and execute skills  Self-efficacy: An individual’s capacity to act effectively to bring about desired results, as perceived by the individual  Perceived behavioural control: an individual’s perception of the ease or difficulty of performing the behaviour of interest  Beliefs: The thing believed; the proposition or set of propositions held true  Self-esteem: The degree to which the qualities and characteristics contained in one’s self concept are perceived to be positive  Empowerment: The promotion of the skills, knowledge, and confidence necessary to take great control of one’s life as in certain educational or social schemes; the delegation of increase decision-making powers to individuals or groups in a society or organization  Professional confidence: An individual’s beliefs in his or her repertoire of skills, and ability, especially as it is applied to a task or set of tasks. | In the context of type 2 diabetes (T2D) screening for women with previous gestational diabetes (GDM), beliefs about capabilities could relate to discussion about:   - a woman’s beliefs about her capability to attend T2D screening - beliefs about own judgement (e.g., continued home glucose monitoring or beliefs in own health perception. (i.e., basing current knowledge on past experience of feeling sick during pregnancy prior to GDM diagnosis). - ease/difficulty to attend T2D screening (e.g., lack of perceived behavioural control) - suggestions to overcome barriers to attending T2D screening - confidence in ability to discuss GDM and T2D screening with healthcare professionals or others - If existing physical or psychological factors prevents participant from attending T2D screening (e.g., needle phobia)   Alternate coding for related material (depending on the context):   - when discussing actual ability to communicate about T2D screening in English as a second language (*skills*) - when discussing confidence in to speak in English to their HCP (*beliefs about capabilities*) |
| 1. Optimism   Confidence that things will happen for the best or that desired goals will be attained | | Optimism: The attitude that outcomes will be positive, and that people’s wishes or aims will be ultimately fulfilled  Pessimism: The attitude that things will go wrong, and that people’s wishes or aims are unlikely to be fulfilled  Unrealistic optimism: the inert tendency for humans to over-rate their own abilities and chances of positive outcomes compared to those of other people | In the context of type 2 diabetes (T2D) screening for women with previous gestational diabetes (GDM), optimism could relate to discussion about:   - personal susceptibility to developing T2D in the future - outcome of T2D screening (e.g., fatalism) - expressing confidence that they are not at risk if T2D (proximal or distal) - feeling healthy and not in need of follow-up   Note: refrain from subjectively judging a participant’s personal risk. |
| 1. Beliefs about consequences   Acceptance of the truth/reality about or validity of outcomes of a behaviour in a given situation | | Beliefs: The thing believed; the proposition or set of propositions held true  Outcome expectancies: Cognitive, emotional, behavioural, and affective outcomes that are assumed to be associated with future or intended behaviour. These assumed outcomes can either promote or inhibit future behaviours.  Characteristics of outcome expectancies: Cognitive, emotional, behavioural, and affective outcomes that are assumed to be associated with future or intended behaviour. These assumed outcomes can either promote or inhibit future behaviours.  Anticipated regret: a sense of the potential negative consequences of a decision that influences the choice made: for example, an individual may decide not to make an investment because of the feelings associated with an imagined loss  Consequents: An outcome behaviour in a given situation | In the context of type 2 diabetes (T2D) screening for women with previous gestational diabetes (GDM), beliefs about consequences could relate to discussion about:   - purpose, value, and effectiveness of T2D screening - beliefs about T2D screening outcomes (including that testing will falsely diagnose T2D) - lack of concern about discovering glucose status (therefore, not motivated to attend screening) - perceived long term outcomes of T2D screening - expressed regret at not attending T2D screening - impact of screening on personal life (e.g., interfere with work, ability to manage life if T2D diagnosis) - material consequences of T2D screening (i.e., what is likely to happen as a result of T2D screening); can be positive or negative - beliefs about severity or consequences of the T2D itself - expectation to sharing GDM diagnosis of GDM with others   Alternate coding for related material (depending on the context):   - How T2D screening will make a woman feel (*emotion*) |
| 1. Reinforcement   Increasing the probability of a response by arranging a dependent relationship, or contingency, between the response and a given stimulus | | Rewards (proximal / distal, valued / not valued, probable /improbable): Return or recompense made to, or received by a person contingent on some performance  Incentives: An external stimulus, such as condition or object, that enhances or serves as a motive for behaviour  Punishment: The process in which the relationship between as response and some stimulus or circumstance results in the response becoming less probable; a painful, unwanted, or undesired event or circumstance imposed as a penalty on a wrongdoer  Consequents: An outcome of behaviour in a given situation  Reinforcement: A process in which the frequency of a response is increased by a dependent relationship or contingency with a stimulus  Contingencies: A conditional probabilistic relation between two events. Contingencies may be arranged via dependencies, or they may emerge by accident  Sanctions: A punishment or other coercive measure, usually administered by a recognised authority, that is used to penalise and deter inappropriate or unauthorized actions | In the context of type 2 diabetes (T2D) screening for women with previous gestational diabetes (GDM), reinforcement could relate to discussion about:   - positive or negative experience of original OGTT (which led to GDM diagnosis) - reward/positive outcome at previous T2D screening - negative outcome from previous T2D screening (including fear of needles) - punishment if they did not attend T2D screening - comments about post-partum abandonment (where a lack of reinforcement impacts prioritisation of screening)   Alternate coding for related material (depending on the context):   - Negative or positive outcome expectancies. (e.g., sweet syrupy solution consumed in OGTT is sickening for some and pleasant for others), *beliefs about consequences* - if existing physical or psychological factors prevent participant from attending screening (e.g., needle phobia), *beliefs about capabilities* |
| 1. Intentions   Conscious decision to perform a behaviour or a resolve to act in a certain way | | Stability of intentions: ability of one’s resolve to remain in spite of disturbing influences  Stages of change model: A model that proposes that behaviour change is accomplished through five specific stages  Trans theoretical model and stages of change: a five-stage theory to explain changes in people’s health behaviour. It suggests that change takes time, that different interventions are effective at different stages, and that there are multiple outcomes occurring across the stages | In the context of type 2 diabetes (T2D) screening for women with previous gestational diabetes (GDM), intentions could relate to discussion about:   - how motivated the woman feels to take part in T2D screening - intention to attend/not attend the 6–12-week Oral Glucose Tolerance Test - intention to attend/not attend the annual T2D screening test - stability of intentions to attend/non attend T2D screening   Alternate coding for related material (depending on the context):   - accuracy of T2D screening methods (*beliefs about consequences*) - priority of screening (*goals*)   Note: do not code reasons for intentions. Instead, focus on statements that directly reflect intention. |
| 1. Goals   Mental representation of outcomes or end states that an individual wants to achieve | | Goals (distal / proximal): desired state of affairs of a person or system, these may be closer (proximal) or further away (distal)  Goal priority: Order of importance or urgency of end state toward which one is striving  Goal / target setting: A process that establishes specific time-based behavioural targets that are measurable, achievable, and realistic  Goals (autonomous / controlled): The end state toward which one is striving: the purpose of an activity or endeavour. It can be identified by observing that a person ceases or changes their behaviour upon attaining this state; proficiency in a task to be achieved within a set period  Action planning: The action or process of forming a plan regarding a thing to be done or a deed  Implementation intention: The plan that one creates in advance of when, where and how one will enact a behaviour | In the context of type 2 diabetes (T2D) screening for women with previous gestational diabetes (GDM), intentions could relate to discussion about:   - achieving T2D screening in the face of competing priorities - goal setting to attend T2D screening - goals they wish to achieve from attending T2D screening (e.g., early identification of T2D so that mitigation can take place) - statements about whether or not T2D screening is a priority - competing goals (e.g., goals which may conflict with T2D screening attendance) |
| 1. Memory, attention and decision processes   The ability to retain information, focus selectively on aspects of the environment and choose between two or more alternatives | | Memory: The ability to retain information or a representation of a past experience, based on the mental processes of learning or encoding retention across some interval of time, and retrieval or reactivation of the memory; specific information of a specific task  Attention: A state of awareness in which the senses are focussed selectively on aspects of the environment and the central nervous system is in a state of readiness to respond to stimuli  Attention control: The extent to which a person can concentrate on relevant cues and ignore all irrelevant cues in a given situation  Decision making: The cognitive process of choosing between two or more alternatives, ranging from the relatively clear-cut to the complex  Cognitive overload / tiredness: The situation in which the demands placed on a person by mental work are greater than a person’s mental abilities | In the context of type 2 diabetes (T2D) screening for women with previous gestational diabetes (GDM), memory, attention and decision processes could relate to discussion about:   - ability to remember when to make appointment for and/or attend T2D screening (proximal and distal) - when/why it would be easy to forget to screen for T2D - description of decision process to attend T2D screening - impact of early parenthood on memory, attention, and ability to make decisions - comments about participant losing referral forms and reminders   Alternate coding for related material (depending on the context):   - emotion-based comments about feeling overwhelmed with care for the newborn/postpartum period/or other life circumstances (*emotion*). |
| 1. Environmental context and resources   Any circumstance of a person’s situation or environment that discourages or encourages the development of skills and abilities, independence, social competence, and adaptive behaviour | | Environmental stressors: External factors in the environment that cause stress  Resources / material resources: Commodities and human resources used in enacting a behaviour  Organisational culture /climate: A distinctive pattern of thought and behaviour shared by members of the same organization and reflected in their language, values, attitudes, beliefs, and customs  Salient events / critical incidents: Occurrences that one judges to be distinctive, prominent, or otherwise significant  Person x environment interaction: Interplay between the individual and their surroundings  Barriers and facilitators: mental, emotional, or behavioural limitations/strengths in individuals or groups | In the context of type 2 diabetes (T2D) screening for women with previous gestational diabetes (GDM), environmental context and resources could relate to discussion about absence or presence of:   - Resources or learnings which have encouraged (or discouraged) the development of skills (e.g., information not in the woman’s first language may be inaccessible) - Quality and preferred material resources (clinic reminders, websites, letters). - Environmental barriers or enablers to attendance (e.g., lack of time/financial resources/access to T2D screening facility/childcare/healthcare professional advice - critical incidents which impacted attendance (or decisions to attend) T2D screening - education and/or information sessions - comments about post-partum abandonment (except where a lack of reinforcement impacts prioritisation of screening, then: *reinforcement*) - timing (e.g., too soon after operative birth)   Alternate coding for related material (depending on the context):   - discussion between health care providers and participants about T2D screening *(social influences*) - when learning about screening from educational event (*Knowledge* or *Skills*, depending upon the context. |
| 1. Social influences   Interpersonal processes that can cause an individual to change their thoughts, feeling or behaviours. | | Social pressure: the exertion of influence on a person or group by another person or group  Social norms: Socially determined consensual standards that indicate a) what behaviours are considered typical in a given context and b) what behaviours are considered proper in the context  Group conformity: The act of consciously maintaining a certain degree of similarity to those in your general social circles  Social comparisons: The process by which people evaluate their attitudes, abilities, or performance relative to others  Group norms: Any behaviour, belief, attitude, or emotional reaction held to be correct or acceptable by a given group in society  Social support: perception or provision of assistance or comfort to others, typically in order to help them cope with a variety of biological, psychological and social stressors. Support may arise from any interpersonal relationship in an individual’s social network, involving friends, neighbours, religious institutions, colleagues, caregivers of support groups  Power: The capacity to influence others, even when they try to resist this influence  Intergroup conflict: Disagreement or confrontation between two or more groups and their members. This may involve physical violence, interpersonal discord, or psychological tension.  Alienation: Estrangement from one's social group; a deep-seated sense of dissatisfaction with one's personal experiences that can be a source of lack of trust in one's social or physical environment or in oneself; the experience of separation between thoughts and feelings  Group identity: the set of behavioural or personal characteristics by which an individual is recognizable [and portrays] as a member of a group  Modelling: In developmental psychology the process in which one or more individuals or other entities serve as examples (models) that a child will copy | In the context of type 2 diabetes (T2D) screening for women with previous gestational diabetes (GDM), social influences could relate to discussion about:   - presence/absence of support from partner/friends/family/cultural group/community groups/wider society (impacting a woman’s ability to attend T2D screening) - Social/cultural expectation about being a mother expressed by others - how others influence T2D screening (including professional advice/recommendation) - Comparison with T2D screening behaviours of other women with prior GDM - Confidence in ability of physician or other health care professionals. - Degree of trust in physician or other health care professionals - Whether a health care provider recommended screening to them   Alternate coding for related material (depending on the context):   - group identity comments coded here, but personal identity comments coded in *Social/Professional Role and Identity* domain. - If resources are social in nature (e.g., family member available to drive patient to appointment), consider double coding with *Environmental context and resources*. |
| 1. Emotion   A complex reaction pattern, involving experiential, behavioural and physiological elements, by which the individual attempts to deal with a personally significant matter or event | | Fear: An intense emotion aroused by the detection of imminent threat, involving an immediate alarm reaction that mobilizes the organism by triggering a set of physiological changes  Anxiety: A mood state characterized by apprehension and somatic symptoms of tension in which an individual anticipates impending danger, catastrophe, or misfortune  Affect: An experience or feeling of emotion, ranging from suffering to elation, from the simplest to the most complex sensations of feelings, and from the most normal to the most pathological emotional reactions  Stress: A state of physiological or psychological response to internal or external stressors  Depression: A mental state that presents with depressed mood, loss of interest or pleasure, feelings of guilt or low self-worth, disturbed sleep or appetite, low energy, and poor concentration  Positive / negative affect: the internal feeling/state that occurs when a goal has/has not been attained. A source of threat has/has not been avoided, or the individual is/is not satisfied with the present state of affairs  Burn-out: Physical, emotional, or mental exhaustion, accompanied by decreased motivation, lowered performance and negative attitudes towards oneself and others | In the context of type 2 diabetes (T2D) screening for women with previous gestational diabetes (GDM), emotion could relate to discussion about:   - Descriptions of emotions experiences when scheduling or attending T2D screening - Include emotions of discomfort and unease regarding screening attendance - experience of GDM diagnosis - fear of ongoing impact of GDM on mother and/or baby - potential for T2D diagnosis / fear of T2D diagnosis - impact of early parenthood on emotions (e.g., lack of energy, stress, lack of sleep)   Alternate coding for related material (depending on the context):   - expectations of negative screening experience, or diagnosis of T2D (*beliefs about consequences*) |
| 1. Behavioural regulation   Anything aimed at managing or changing objectively observed or measured actions | | Self-monitoring: A method used in behavioural management in which individuals keep a record of their behaviour, especially in connection with efforts to changes or regulate the self; a personality trait reflecting an ability to modify one’s behaviour in response to a situation  Breaking habit: to discontinue a behaviour or sequence of behaviours that is automatically activated by relevant situational cues  Action planning: The action or process of forming a plan regarding a thing to be done or a deed. | In the context of type 2 diabetes (T2D) screening for women with previous gestational diabetes (GDM), behavioural regulation could relate to discussion about steps taken to provide or use:   - Self-regulatory strategies mentioned in relation to T2D screening attendance (any strategy that helps the person to enact their intention to go for screening) - managing activities to **prioritise** T2D screening (even if they don’t actually attend) |

# S6 Preferences for resources and reminders, and alternatives to the 6-12-week OGTT

## Participant Diabetes Screening Reminder Format Preferences

Participants were asked about their preferences and suggestions for resource and reminder delivery format. Preferences for format and delivery of resources and reminders were evenly distributed across paper, email and SMS based mediums followed by online, app or ICS file formats. These findings suggest resources and reminders should be provided in a range of formats to address individual needs and preferences.

**Preferences for Resource and Reminder Format**

| **Format** | **Preference** | **Exemplar quotes** |
| --- | --- | --- |
| Paper (letter or pamphlets) | 7 (37%) | “I do think that a letter and a physical reminder that I can stick on my fridge and have it in my face is probably going to be a better reminder to get it done” (ID05) |
| Email | 6 (32%) | “Like, emails now are probably a lot simpler. I was getting letters or pamphlets, and I flipped through them… I don’t take a lot of that in” (ID04) |
| Text | 6 (32%) | “…text works quite well for me” (ID09) |
| Online | 4 (21%) | “… if the kid needs a feed and it’s, you know, 2:00 in the morning, what do you do? You’re just, like, scrolling through ((laughs))” (ID01) |
| App | 2 (11%) | “I was just going to add quickly, I don’t know if this could tie into it, but I’ve got like a vaccination app for my son in terms of like when the vaccinations are due. And it sends – like I get pop ups” (ID12) |
| Calendar invite (ICS file) | 1 (5%) | “…if there was some way or like sending out a – a – like an ICS file – it’s like you can put this in your calendar because you need to have done it by this time and you do it straight away when you get it” (ID05) |

*Note.* Data are n (%)

## Participant resource and reminder content suggestions and preferences

Participants were also asked about their preferences for resource content. Many participants communicated a preference for resources that are brief and include infographics to facilitate easy processing of the information. Some also expressed that inclusion of a strong rationale including outlining potential consequences of undiagnosed diabetes would motivate screening attendance. Conversely, one participant expressed an aversion to fear-based messaging and indicated a preference for more empowering content that outlines the positives aspects of screening and receiving a diabetes diagnosis.

| **Content suggestion** | **Exemplar quotes** |
| --- | --- |
| Include strong rationale | “So, for me, if I was to make improvements, I would use really simple English to explain the – ‘why’ it’s a good idea” (ID03) |
| Brief/straightforward | “…short and the font is around, 18, ((laughs)), say- you’ve been reminded to do your annual diabetes screening” (ID06) |
| Include infographics/images | “An infographic… I think that would be a more succinct way of presenting the information so that you can digest it quickly” (ID05) |
| Inclusion of timeline | “I think a timeline of, you know, ‘By this date you should’ve had this done.’ I think that would’ve been helpful, 'cause the days kind of blur into each other when you have a newborn” (ID13) |
| Risk warning (for) | “… it would’ve been higher in priorities if the warnings – if it highlighted what can go wrong– if you have type 2 diabetes. Like, on smoking packets – that’s a really extreme example, but seeing the visuals and seeing a reminder of, ‘Oh. Okay. We really don’t want to have this or to go there’” (ID07) |
| Risk warning (against) | “I would say, positive outcomes …it’s all negative, right – so it’s not, like, oh, when – you – got diabetes – you get these – you might pass it to your children and – and you might have this, and then down the track you will get this, down the track you will get that….– if I get information about – positive side of it, say, – if you get diabetes, the positive impact would be this” (ID06) |

## Participant preferences to alternatives to the current OGTT

Due to temporary changes in screening guidelines during the COVID-19 pandemic, participants were asked about opinions on home-based alternatives to the OGTT. Most endorsed the suggestion, noting that it would broadly be a more convenient and comfortable option that would allay concerns about contracting COVID-19 in the screening environment. Some, however noted concerns about the accuracy of home-based alternatives, whether they would have the skills perform the procedure and emotional response if they were to receive their screening results on their own.

| **Response type** | ***N* (%)** | | **Theme** | **Exemplar quote** |
| --- | --- | --- | --- | --- |
| Endorsed alternative | 14 (74%) | Convenience | | “Um, having flexibility to do things at home when you're a Mum is awesome” (ID08) |
|  |  | Physical safety | | “I would be much more happy to do – to do that kind of process. And I think given that the doctor situation with COVID at the moment” (ID12) |
|  |  | Comfort | | “…if I had the option of doing something at home, I would’ve much preferred that, because in my situation where I woke up at 5:00AM and then had to wait until the clinic opened, if it was a fasted one, I could’ve just done that in my own time and gotten on with my day… it might have made it more comfortable” (ID21) |
| Expressed concerns | 9 (47%) | Concerns about skills/ performing home testing correctly | | “It would be interesting if you can get a consistent result with it, because when you're at home you tend to be running around a bit more….So, it’s, sort of, easier being out of the house to sit there for two hours, than it is if you're at home, you’re, like, far more tempted to do other things because you're time poor” (ID19) |
|  |  | Concerns about receiving result on one’s own | | “…in that, sort of ten-week period where I was monitoring, I had three high readings, and I was just devastated, because I thought, “What have I done wrong? I’ve eaten what I should be eating, I’ve exercised when I should have, I don’t understand why the reading was high. Um, but yeah…I’d be upset” (ID11) |
|  |  | Concerns about inaccuracy | | “…if I’m going to do it and it’s going to be wrong, or not – inaccurate reading, then I might as well go and do in one go, and I don’t need to keep repeating it” (ID06) |
|  |  | Concerns about technology | | “…this is too much technology that – in my mind” (ID06) |

# S7 TDF domains of ‘less’ importance

Eight of the 14 TDF domains were represented in at least two of the three importance criteria and classified as ‘high’ importance (see in-text Table 2). This section summarises five TDF domains classified as ‘less’ importance, by key sub-themes. One remaining TDF domain (Skills) was not represented.

## TDF domain: Behavioural regulation

*Theme: Proactive behaviour****.***  Some participants engaged in proactive behaviour or implemented self-regulatory strategies to facilitate their attendance to screening. For example, when there was absence of prompting or communication from health professionals to attend postpartum diabetes screening, some participants initiated this conversation themselves*: “It was just my own initiative”* (ID01). Others implemented regulatory strategies such as personal prompts to facilitate attendance to postpartum diabetes screening: *“I kept the referral form from the obstetrician on the fridge, so it was a reminder”* (ID21). For some, difficulty with initiating behaviours necessary to attend diabetes screening was a barrier: *“I should really get the script and do all that, but, no, I didn’t”* (ID06).

## TDF domain: Optimism

*Theme: Perceived personal risk.* Most participants perceived themselves as being at risk of developing type 2 diabetes to some degree. Believing oneself to be at an increased risk of developing T2D motivated increased attention to managing health for some: *“I know that I'm in a high-risk category now. So, um – like, I know that that’s a real possibility, and that’s probably why I'm a little bit more conscious about, like, the food and the exercise”* (ID04).

Participants who described additional risk factors for T2D such as a family history of diabetes, pre-existing health issues or a previous diagnosis of prediabetes often perceived their risk to be high: *“Mum and my grand Mum from Mum’s side, they both have high blood sugar… I believe there’s a gene”* (ID09).

*“I know polycystic ovarian syndrome can already put you in the, ah, higher risk category because of insulin resistance and what goes along with that*” (ID10)

One participant felt their risk was not immediate*: “I think there is a fair chance, realistically speaking, that I probably might end up with diabetes, but I think that’s going to be quite a few years away”* (ID01). For others, perceived risk was highest following pregnancy and subsequently decreased following attendance to the postpartum OGTT and receiving a negative result *“…in my mind, it sort of went to the back. I thought, ‘Okay. Phew. The test was – came back, um, negative. So, all good’”* (ID17).

## TDF domain: Intentions

*Theme: Intention to screen.* Most participants indicated high intention to continue to attend diabetes screening: “Yes, I definitely will go” (ID15).

Most described diabetes screening as a high priority “…it would certainly be, sort of, fairly high up on my list to book it in” (ID16). However, several participants indicated that while they intended to continue being screened for diabetes, they would not be likely to attend frequently or seek out an appointment:

*“Maybe not every – every year. Maybe every two years”* (ID09).

*“I might not go specifically for that, 'cause it doesn’t seem like it’s a emergency thing, but, the next time I'm at the doctor’s I would probably ask for a referral at the same time”* (ID07)

## TDF domain: Goals

*Theme: Goal priority.* Desire to manage T2D risk, to maintain health to care for children and desire to become pregnant in the future were goals that enabled postpartum diabetes screening.

*“…my health is something I want to maintain, especially keeping up with five kids*” (ID10)

*“If I want to have another child one day, then obviously I’d want to make sure that I knew exactly what my body was doing at that time”* (ID04)

A barrier to achieving screening goals was competing demands:

*“…it was something that I was worried about, but in comparison to everything else it was very much at the bottom of the list”* (ID21)

*“We’re trying to buy a house, so I need to try and make sure that all my shifts are regular and that I’m not taking sick days and stuff like that”* (ID02)

## TDF domain: Reinforcement

*Theme: Previous experience with OGTT.* For some participants, negative experiences during the antenatal OGTT were barriers to attending postpartum diabetes screening. These included feeling unwell, a strong aversion to the glucose solution and injury from blood testing:

*“I just felt really sick from, I gather, the amount of sugar that was in me”* (ID19)

*“I was a bit, like, thinking I'm going to have to have this vile drink”* (ID25)

*“I was also scared, because the first time I did it, um, the person, it hurt a lot”* (ID17)

## TDF domain: Skills

No data
